# Bystander cells enhance NK cytotoxic efficiency by reducing search time

**DOI:** 10.1038/srep44357

**Published:** 2017-03-13

**Authors:** Xiao Zhou, Renping Zhao, Karsten Schwarz, Matthieu Mangeat, Eva C. Schwarz, Mohamed Hamed, Ivan Bogeski, Volkhard Helms, Heiko Rieger, Bin Qu

**Affiliations:** 1 Biophysics, Center for Integrative Physiology and Molecular Medicine, School of Medicine, Saarland University, 66421 Homburg, Germany; 2 Department of Theoretical Physics, Saarland University, 66123 Saarbrücken, Germany; 3 Center for Bioinformatics, Saarland University, 66041 Saarbrücken, Germany; 4 Institute for Biostatistics and Informatics in Medicine and Ageing Research, Rostock University Medical Center, 18057 Rostock, Germany

## Abstract

Natural killer (NK) cells play a central role during innate immune responses by eliminating pathogen-infected or tumorigenic cells. In the microenvironment, NK cells encounter not only target cells but also other cell types including non-target bystander cells. The impact of bystander cells on NK killing efficiency is, however, still elusive. In this study we show that the presence of bystander cells, such as P815, monocytes or HUVEC, enhances NK killing efficiency. With bystander cells present, the velocity and persistence of NK cells were increased, whereas the degranulation of lytic granules remained unchanged. Bystander cell-derived H_2_O_2_ was found to mediate the acceleration of NK cell migration. Using mathematical diffusion models, we confirm that local acceleration of NK cells in the vicinity of bystander cells reduces their search time to locate target cells. In addition, we found that integrin β chains (β1, β2 and β7) on NK cells are required for bystander-enhanced NK migration persistence. In conclusion, we show that acceleration of NK cell migration in the vicinity of H_2_O_2_-producing bystander cells reduces target cell search time and enhances NK killing efficiency.

Natural killer (NK) cells play a key role in eliminating virus-infected or tumorigenic cells without prior exposure to antigen for their activation^1^,^2^. The interaction between MHC class I molecules on target cells and NK inhibitory receptors plays a major role in regulating NK cell activation. Down-regulated expression of MHC class I molecules on pathogenic cells, following infection by certain virus or neoplastic transformations, renders those cells susceptible to NK cell attack^3^,^4^,^5^. Upon recognition, NK cells form a tight junction with a target cell, which is called immunological synapse (IS)^6^. Lytic granules (LG) containing perforin and granzymes are then deployed, which constitutes the major mechanism to induce target death^7^. Upon IS formation, LG are accumulated and released exclusively at the IS to avoid damage of surrounding non-target bystander cells^6^.

NK cells constantly patrol peripheral organs as essential effectors of immune surveillance^8^. NK cells can be rapidly recruited to inflammatory sites^9^ and infiltrate into tumors^10^. Gradients of chemokines are favorable as directional cues to guide immune cells^11^ towards or away from anatomically stable structures such as lymphatic vessels^12^ or bone marrow^13^. NK cell trafficking and recruitment are mainly regulated by G-protein coupled chemotactic receptors^8^,^14^. Extracellular messengers, such as reactive oxygen species (ROS), could also play a role to guide NK cells to their destination. Previous studies have shown that hydrogen peroxide (H_2_O_2_), a relatively stable form of ROS, can recruit leukocytes to wounded sites^15^ or oncogene-transformed cells^16^.

In a pathological scenario, not all cells in a given NK-patrolling area are necessarily target cells. For example, NK cells encounter stromal cells^17^, infiltrated immune cells^18^ as well as malignant cells with expression of MHC class I molecules. These bystander cells pose a challenge to NK cells to efficiently identify their targets in a complex microenvironment. Whether and how the presence of bystander cells can affect the efficiency for NK cells to find and kill their targets has not yet been investigated.

In this study we show that the presence of non-target bystander cells unexpectedly enhanced the killing efficiency as well as NK cell migration. The presence of bystander cells accelerates NK cell migration via H_2_O_2_. We establish three mathematical diffusion models and confirmed that local acceleration of NK cells in the presence of bystander cells can decrease search time, and thus increase killing efficiency. We also show that the surface molecule β-integrin on NK cells is involved in mediating bystander-enhanced NK persistence. Together, our findings unravel a novel regulation mechanism between the microenvironment and NK cells.

## Results

### Presence of bystander cells increases killing efficiency and enhances NK cell migration

**Figure 1:**
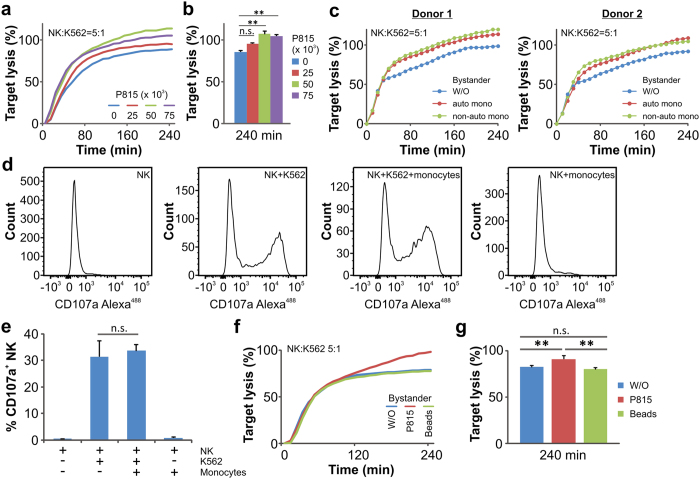
The presence of bystander cells increases NK cell-mediated cytotoxicity. (**a,b**) Target lysis in the presence of bystander cells is analyzed by the real-time killing assay. K562 cells were used as targets for primary human NK cells with an effector to target (E:T) ratio of 5:1. Target cells were plated at 25 × 10^3^ cells/well. P815 cells were used as bystanders at a density of 25 × 10^3^, 50 × 10^3^ or 75 × 10^3^ cells/well, respectively. One representative donor is shown in (**a**). Quantification from three donors is shown in (**b)**. (**c**) Bystander primary monocytes enhance NK killing efficiency. Autologous monocytes (auto mono) and NK cells were isolated from the same donors. Non-autologous monocytes (non-auto mono) were from the other donor. K562 and NK cells were plated as described in **a**. Monocytes were plated at a density of 75 × 10^3^ cells/well. (**d,e**) The presence of monocytes does not enhance LG release upon target recognition. Primary monocytes and NK cells are from the same donors. The density of monocytes, NK and K562 cells were the same as described in **c**. Samples were incubated at 37 °C with 5% CO_2_ for 4 hours before flow cytometry analysis. Quantification from four donors is shown in (**e**). One representative donor is shown in (**d**). (**f,g**) Cell-sized beads do not enhance NK cell killing. K562 cells were used as targets (25 × 10^3^ cells/well) for primary NK cells with an E:T ratio of 5:1. Bystander P815 cells or beads were plated at a density of 75 × 10^3^ cells/well. One representative donor out of four is shown in (**f**). Quantification from four donors is shown in (**g**).

We first hypothesized that in the presence of bystander cells, NK cells would require more time to identify their pathologic target cells, due to the need for NK cells to examine each cell they encounter. This in turn should result in an overall reduced killing efficiency. To test this, we used a real-time killing assay, where the cells of interest, normally target cells, were fluorescently labeled with calcein. When target cells are killed by primary NK cells, calcein is released into the supernatant, resulting in a reduction in fluorescence intensity^19^. We first used P815 cells as bystander cells. Unexpectedly, the presence of P815 cells increased rather than decreased the efficiency of target cell lysis by NK cells (Fig. 1a,b). We further confirmed that P815 cells did not trigger NK killing, with (Supplementary Fig. 1a, P815 as bystanders) or without the presence of target cells (Supplementary Fig. 1a, P815).

To test whether the bystander-enhanced NK killing is restricted to P815 cells or indeed a more general mechanism, we used primary human peripheral monocytes from the same donor (autologous) or from a different donor (non-autologous) as bystanders. As shown in Fig. 1c, both autologous and non-autologous monocytes increased the killing efficiency of primary NK cells. Furthermore, we tested human umbilical vein endothelial cells (HUVEC) as bystander cells. Results from three donors show that the presence of HUVEC increased NK killing efficiency substantially in two donors (Donor 1 and 3) and marginally in one donor (Donor 2) (Supplementary Fig. 2). These findings suggest that bystander-enhancement of NK cell killing efficiency is a general mechanism.

To pinpoint the mechanisms whereby bystander cells enhance NK cell killing, we first examined whether bystander cells increase lytic granule (LG) release of NK cells. LG express the lysosome-associated membrane protein 1 (LAMP1), also known as CD107a, on their membranes. Following target cell recognition by NK cells, lytic granules are released at the immunological synapse resulting in the integration of CD107a into the plasma membrane of NK cells. CD107a is widely used as a functional marker to identify LG release in NK cells^20^. We found that the surface staining of CD107a on NK cells triggered by target cells was unchanged in the absence or presence of monocytes (Fig. 1d,e) or P815 (Supplementary Fig. 1b,c), indicating that bystanders do not change LG release.

We next investigated whether space occupancy played a role in bystander-enhanced NK killing. For this purpose, we used cell-sized polystyrene beads to replace live bystander cells. The results from our real-time killing assay shows that unlike bystander cells, the polystyrene beads do not enhance the killing efficiency of NK cells (Fig. 1f,g).

In sum, we conclude that the bystander effect on NK cell-mediated cytotoxicity is not a result of increased LG release from NK cells or the mere space occupancy by bystander cells.

**Figure 2:**
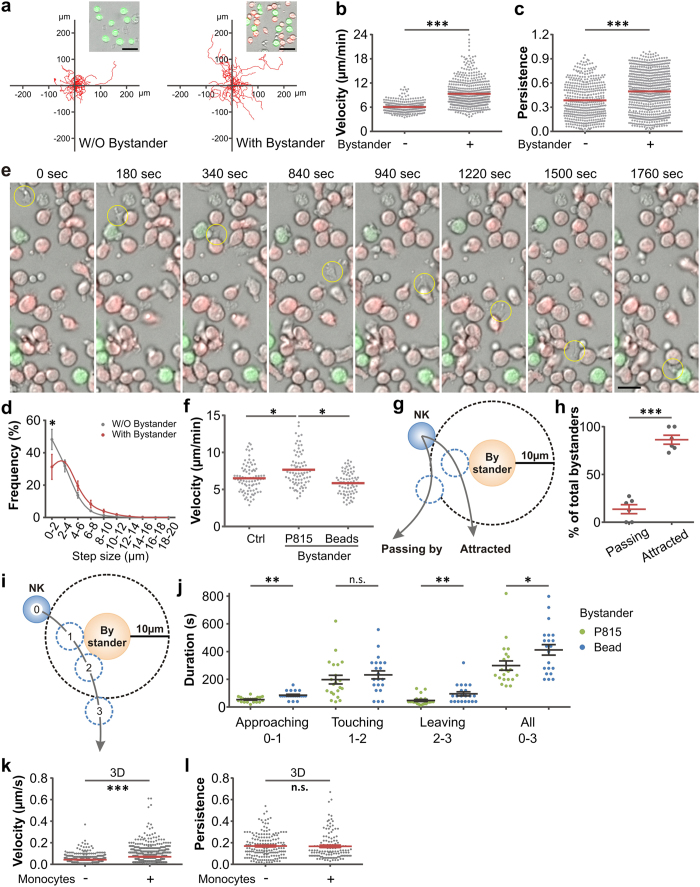
NK cell migration is increased by the presence of bystander cells. (**a–e**) Primary human NK cell migration on coverslips was visualized with the cell observer at 37 °C. Target K562 cells and bystander P815 cells were labeled with calcein-AM (green) and calcein-red-AM (red), respectively. Trajectories of NK cells were tracked manually and 20 randomly chosen trajectories are shown in (**a**). Velocity (**b**), persistence (**c**) and step sizes of killer cell migration (**d**) were analyzed. Results are from three donors. One exemplary migrating NK cell is marked by the yellow circle in **e**. Scale bars are 20 μm. (**f**) Cell-sized beads do not change migration velocity of NK cells. Migration of primary human NK cells on coverslips was visualized with the cell observer at 37 °C. Target K562 cells were loaded with calcein-AM. Beads or calcein-red-AM loaded P815 cells were used as bystanders. NK cells were plated with K562 cells only (Ctrl), K562 and P815 cells (P815) or K562 and beads (Beads). Results are from three independent experiments. (**g,h**) NK cells are attracted to bystander cells. The probability that NK cells bypassed or were attracted to nearby (closer to 10 μm) bystander cells was analyzed as shown in (**g**). To avoid bias, only NK cells that encountered at least 8 bystander cells were analyzed. Quantification from six NK cells is shown in (**h)**. (**i,j**) NK cells are accelerated in the vicinity of bystanders. As depicted in (**i**), NK cells attracted to bystanders (P815 or beads) were analyzed for the duration of approaching (0–1), touching (1–2), leaving (2–3) and full trajectory from approaching to leaving (0–3). Quantification from three experiments is shown in (**j**). **(k,l)** Lightsheet microscopy was used to analyze NK cell migration in 3D at 37 °C. Migration velocity is accelerated by bystander cells. For imaging, primary NK cells, target K562 cells and monocytes were loaded with CellTracker™ Deep Red, Calcein-AM and CellMask™ Orange, respectively. Matrigel was polymerized at 37 °C with 5% CO_2_ for 30 minutes before imaging. NK cells were automatically tracked and analyzed by Imaris. Results are from two donors.

Next we examined migration properties of NK cells, which are relevant for efficient NK cell search time to localize target cells and thus influence the killing efficiency. Target cells (K562) and bystander cells (P815) were distinguished by calcein-green and calcein-red, respectively (Fig. 2a, inset). To avoid any possible interference of NK cell functions by a fluorescent dye, we tracked NK cells using bright field with no cell staining. Within the same time frame, NK cells in the presence of bystanders migrated further away from their starting point (Fig. 2a). Our analyses show that the presence of bystander cells significantly enhanced NK cell velocity (Fig. 2b) as well as NK persistence (Fig. 2c), defined as the displacement between the start and end point divided by total length of the trajectory. Quantification of the step size of NK cells revealed higher values in the presence of bystander cells (Fig. 2d). We also found that NK cell do not avoid bystander cells, in fact they appear to approach bystanders on their path to finding target cells (Fig. 2e and Supplementary Movie 1). In contrast, cell-sized polystyrene beads did not enhance NK migration velocity (Fig. 2f). These findings suggest that the increase in target eradication by NK cells in the presence of bystander cells is likely induced by accelerated and more persistent NK cell migration.

We further quantified how often NK cells were attracted to bystander cells using the analysis shown in Fig. 2g. When an NK cell approaches a bystander cell (at 10 μm distance), this NK cell can either make contact with this bystander (defined as ‘attracted’) or pass by without contact (defined as ‘passing by’). We found that in most cases (more than 80%) NK cells were attracted to bystander cells (Fig. 2h).

To investigate whether bystander cells influence NK cell migration locally, we analyzed the velocity of NK cells in the vicinity of bystander cells. As shown in Fig. 2i, the trajectory of an NK cell near a bystander was separated into three phases: approaching (0–1), touching (1–2) and leaving (2–3). Using this classification we compared P815 cells and cell-sized polystyrene beads as bystanders. NK cells did not distinguish between P815 and the beads regarding the contact phase, indicating that NK cells need comparable times to examine different objects. On the other hand, approaching and leaving times were clearly accelerated in the case of P815 bystanders compared to the beads (Fig. 2j). Adding up the times needed for approaching, touching and leaving, NK cells altogether need less time to examine P815 bystanders compared to beads (Fig. 2j, All). This is consistent with an increased migration velocity in the vicinity of P815 bystanders. We conclude that bystander cells attract NK cells and locally accelerate NK cell migration.

In most cases, *in vivo* NK cells migrate in a three dimensional (3D) environment. We thus examined by lightsheet microscopy whether NK cell migration in 3D matrices could be affected by the presence of bystander cells. Primary human NK cells and monocytes were isolated from the PBMCs of the same healthy donors. We observed that the velocity of NK cells was enhanced by the presence of bystander monocytes, whereas the persistence of NK cells was not altered (Fig. 2k,l). In conclusion, NK cells are also accelerated by bystander cells in a 3D environment. Different from the 2D scenario, however, in 3D collagen matrices the persistence of NK cells was not affected by the bystander cells.

### The increased motility of NK cells near bystander cells decreases search time for target cells

Considering the results described so far, we hypothesized that the local acceleration of NK cells close to bystanders would decrease search time to locate target cells and thereby enhance NK killing efficiency. We tested this hypothesis using a mathematical model for the random search process performed by the NK cells in a two-dimensional environment. The model consists of: 1) N_k_ disk-like particles performing random Brownian motion with diffusion constant D in a plane of size L × L (representing the NK cells searching for target cells), 2) N_t_ disk-like target cells which are placed at random positions in the plane and do not move (target cells also barely move in our *in vitro* experiments), and 3) the number of remaining target cells as a function of time, N_t_(t). Whenever a killer disk touches a target disk, the target disk is removed and N_t_(t) decreases by one. Moreover, the model contains N_o_ randomly placed immobile disk-like obstacles of radius r_obs_ representing inactive NK cells. Their effect is only to exclude areas where the killers can go. Finally, the model contains N_b_ randomly placed immobile disk-like particles with radius R representing bystanders. There are two effects of bystander cells on the motion of the NK cells in the model: 1) they exclude the area occupied by the bystander itself, and 2) they change the diffusion constant for the killers from D to D_acc_ > D in a circular region of radius Δ > R around the center of the bystander cell. The increased diffusion constant D_acc_ mimics the experimentally observed acceleration of the killer motion close to the bystander cells.

With the help of computer simulations (see Materials and Methods) we calculated for various parameter sets (N_k_, N_t_(t = 0), N_o_, N_b_, r_obs_, D_acc_, Δ): 1) the average number of surviving targets at time t, <N_t_(t) >, and 2) the average half-time t_1/2_, the average time at which half the targets were found and removed. The average half time (t_1/2_) is a measure for the killing efficiency of the NK cells in this model framework with the given parameters. For the model calculations, it is convenient to use dimensionless units; we used the diameter of the NK cells as the unit of length, i.e. set R = 0.5, and fixed the time unit by setting the diffusion constant of the NK cells to D = 1. Moreover, we fixed the system size to L = 50 and chose the particle numbers such that particle densities in the model match the cell densities in the experiments.

**Figure 3:**
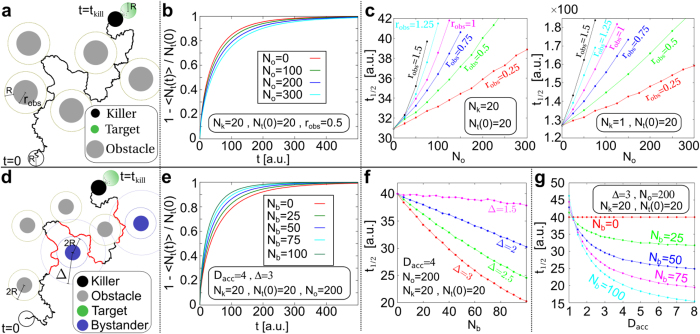
Local acceleration of NK cells by bystander cells reduces NK cell search time. (**a**) Sketch of the mathematical model of a Brownian (diffusive) disk-like particle performing a random search for disk-like targets among disk-like obstacles: the black disk represents one randomly moving NK cell, the black wiggly lines its diffusive motion with diffusion constant D, grey disks the immobile obstacles of radius r_obs_, and green disks immobile targets of radius R. Targets that are hit, here at time t_kill_, are removed upon contact with the NK cells. (**b**) The ratio of killed targets is depicted as a function of time for N_k_ = 20 killers, N_t_(0) = 20 targets, different numbers of obstacles N_o_, and r_obs_ = 0.5. (**c**) The average half time t_1/2_ is given as a function of the number of obstacles N_o_ for different values of r_obs_ for the same number of killers and targets (left) and for the case of only one killer (right). (**d**) Same as (**a**) but now with bystanders (blue). Diffusion of the NK cells is accelerated by an increased diffusion constant, D_acc_ > D, within the circles with radius Δ around bystanders, as indicated by red portions of the wiggly lines representing the random killer motion. Otherwise the symbols are similar to (**a**). (**e**) The ratio of killed targets for the model shown in (**d**) as a function of time for N_k_ = 20 killers, N_t_(0) = 20 targets, N_o_ = 200 obstacles, D_acc_ = 4 and Δ = 3 and different numbers of obstacles. **(f)** Average half time t_1/2_ as a function of N_b_ for N_k_ = 20 killers, N_t_(0) = 20 targets, N_o_ = 200 obstacles and D_acc_ = 4 and different values of Δ. (**g**) Average half time t_1/2_ as a function of D_acc_ for N_k_ = 20 killers, N_t_(0) = 20 targets, N_o_ = 200 obstacles and Δ = 3 and different numbers of obstacles.

First we analyzed a scenario that would arise if bystanders were obstacles with no accelerating effect on NK cells (formally: N_b_ = 0). The corresponding model is shown in Fig. 3a. Note that the center of the randomly moving NK cells (black) cannot approach the obstacles (grey) closer than the concentric circle surrounding them. Figure 3b shows the fraction of killed targets up to time t, 1-<N_t_(t) >/N_t_(t = 0), as a function of time for different obstacle numbers N_o_ of radius r_obs_ = 0.5 and using a fixed number of killer and target cells N_k_ = N_t_(t = 0) = 20. The model suggests that the higher the number of obstacles the slower the killing rate. This observation is precisely quantified by the average half-time t_1/2_ as a function of the number of obstacles N_o_ for different obstacle radii r_obs_, once for 20 killers (left) and once for only one killer (right) (Fig. 3c): t_1/2_ increases monotonically with N_o_, and the larger the obstacles the faster NK cells kill. Consequently, bystanders in the experiments cannot simply be considered passive obstacles.

Next, we studied the effect of bystanders accelerating the NK cells in their proximity, represented in the model by N_b_ > 0 as depicted in Fig. 3d. Note that the red parts of the killer trajectory lie within a circle of radius Δ > R around a bystander, where the random motion of the killer is governed by a larger diffusion constant D_acc_ > D. To limit the parameter space, we fixed r_obs_ = R here. Figure 3e shows our results for the fraction of killed targets up to time t, 1- < N_t_(t) >/N_t_(t = 0), as a function of time for different bystander numbers N_b_ and a fixed number of killer and target cells N_k_ = N_t_(t = 0) = 20 and obstacles N_o_ = 200. The increased diffusion constant is D_acc_ = 4 and the acceleration radius Δ = 3. The data demonstrate that the higher the number of bystanders, the faster the killing proceeds. Furthermore, the half-time t_1/2_ is depicted as a function of the number of obstacles N_b_ for different acceleration radii Δ and fixed N_k_, N_t_, N_o_, and D_acc_ (Fig. 3f). Now t_1/2_ decreases monotonically with N_b_, and the larger the acceleration radius the faster NK cells kill. Figure 3g shows that t_1/2_ also decreases monotonically with the diffusion constant D_acc_.

Finally, we examined the robustness of our results using two different discrete lattice-based “random walk” models. In the discrete Model 1, the bystanders are placed in this lattice and all cells could move (Supplementary Fig. 3a). In the discrete Model 2, the bystanders are always immobile and placed between the lattices (Supplementary Fig. 3b). In both discrete models the hopping rates of NK cells are dependent on the neighboring cells. Different combinations for the motility of the cell types in Model 1 (Supplementary Fig. 3c) and various hopping possibilities of NK cells in Model 2 (Supplementary Fig. 3d) were investigated. From both Models, the half time t_1/2_ decreases with an increase of bystander cell numbers (Supplementary Fig. 3c and d). The discrete Model 1 also predicts that the more bystander cells, the faster target cells are found by NK cells (Supplementary Fig. 3e) and the half time t_1/2_ as a function of q (hopping rate to an allowed direction, details see Supplementary Method) decreases with an increase of bystander cell numbers (Supplementary Fig. 3f). Thus, both the continuous model and the discrete models predict that accelerating NK cells by bystanders enhances killing efficiency.

### Bystander cell-generated H_2_O_2_ plays a key role in the acceleration of NK cell migration and enhanced killing efficiency

**Figure 4:**
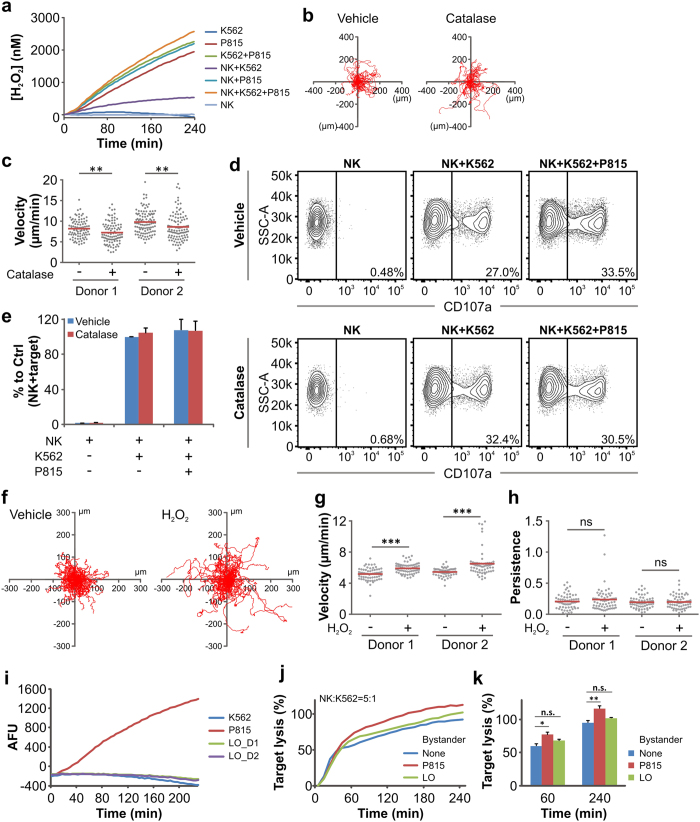
Hydrogen peroxide regulates bystander-mediated increase in NK cell migration. (**a**) H_2_O_2_ production by NK, K562 and P815 cells. As for real-time killing assay, target K562, bystander P815 cells and primary NK cells were plated at a density of 25 × 10^3^, 75 × 10^3^ and 25 × 10^4^ cells/well, respectively. Production of H_2_O_2_ was determined by the Amplex UltraRed assay at 37 °C every 4 minutes for 4 hours. (**b,c**) Depletion of H_2_O_2_ by catalase attenuates bystander cell-enhanced migration velocity of NK cells. NK cell migration on coverslips was visualized at 37 °C using a 10× objective. Primary NK cells were incubated with target K562 cells and bystander P815 cells with or without catalase (10 units/ml). Randomly chosen NK trajectories are shown in (**b**). NK velocity is shown in (**c**). (**d,e**) Depletion of H_2_O_2_ by catalase does not affect degranulation. Primary NK, target K562 and bystander P815 cells were plated at a density of 12.5 × 10^4^, 25 × 10^3^ and 75 × 10^3^ cells/well, respectively. Samples were incubated at 37 °C with 5% CO_2_ for 4 hours before flow cytometry analysis. One representative donor out of five is shown in (**d**). Quantification of all five donors is shown in (**e**). (**f–h**) Addition of H_2_O_2_ enhances velocity but not persistence of NK cell migration. NK cell migration in the presence of vehicle or H_2_O_2_ (2 μM) was visualized at 37 °C with the cell observer. Trajectories, velocity and persistence of NK cell migration are shown in (**f**,**g** and **h**), respectively. (**i**) NK population-depleted PBMCs (LO) produces minimal amount of H_2_O_2_. Bystander cells (P815 or LO) and target K562 cells were plated at 75 × 10^3^ and 25 × 10^3^ cells/well, respectively. Production of ROS was measured by the Amplex UltraRed assay. Arbitrary fluorescence units (AFU) were assigned following the removal of fluorescence background from the medium. Average AFU of triplicates is shown. (**j,k**) NK population-depleted PBMCs (LO) do not increase NK killing efficiency. The real-time killing assay was used to determine target lysis. Bystander cells (P815 or LO) and target K562 cells were plated at 75 × 10^3^ and 25 × 10^3^ cells/well, respectively. One representative experiment is shown in (**j**). Quantification from four experiments is shown in (**k**).

We next searched for molecular mechanisms responsible for accelerating NK cells in the vicinity of bystander cells. We tested H_2_O_2_, which has been shown to influence immune cell migration^21^,^22^,^23^. Analyzing H_2_O_2_ production by different bystander cell types, we found that P815 bystander cells (Fig. 4a) and primary monocytes (Supplementary Fig. 4) but not K562 target cells or NK cells themselves produced H_2_O_2_ (Fig. 4a). To test if environmental H_2_O_2_ is relevant for the bystander effect seen for NK cell migration, we applied the naturally occurring H_2_O_2_ scavenger catalase, which depletes H_2_O_2_ (as confirmed in Supplementary Fig. 5). We then analyzed NK cell migration in the presence of P815 bystander cells with or without catalase. The bystander cell-induced increase in NK cell migration velocity was inhibited by catalase (Fig. 4b,c), whereas LG release of NK cells was not affected (Fig. 4d,e), suggesting that H_2_O_2_ acts specifically on migration but not LG release. We conclude that bystander-produced H_2_O_2_ accelerates NK cell migration and thereby enhances NK killing efficiency.

To further verify if the observed bystander-effects in target killing are due to increased H_2_O_2_, we added 2 μM of external H_2_O_2_ into the growth medium, which is in the range of H_2_O_2_ produced by P815 bystander cells *in vitro* (Supplementary Fig. 6). We found that 2 μM H_2_O_2_ specifically increased the velocity of NK cells (Fig. 4f,g), whereas migration persistence was not affected (Fig. 4h). LG release was also not changed by the addition of H_2_O_2_ (Supplementary Fig. 7), confirming the specific action of H_2_O_2_ on NK cell migration. To further test the importance of H_2_O_2_ for the bystander-enhanced NK killing, we applied NK population-depleted PBMC (LO) as bystander cells, which produce very low levels of H_2_O_2_ (Fig. 4i). P815 cells were used as positive controls. Real-time killing assay analysis shows that NK population-depleted PBMC could not enhance NK cell killing efficiency (Fig. 4j,k). Taken together, these data suggest that the alteration of H_2_O_2_ in the microenvironment generated by bystander cells is responsible for accelerating NK cell migration and the enhanced killing efficiency.

### Beta-integrins on NK cells are responsible for the bystander-enhanced NK cell persistence

**Figure 5:**
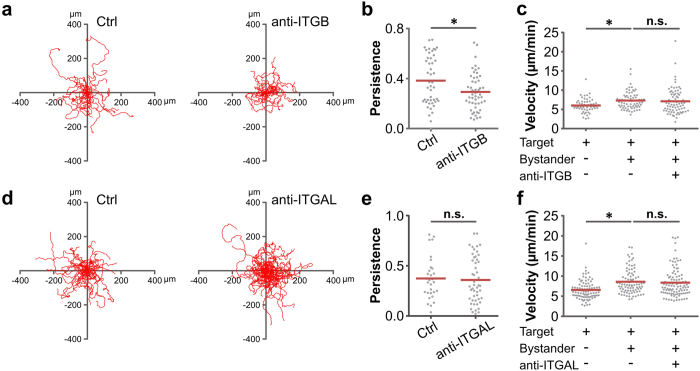
Bystander-enhanced NK cell persistence is dependent on NK cell surface molecule integrin β chains. Primary NK cell migration was visualized at 37 °C by the cell observer with a 20× objective every 20 seconds for 30 min. K562 and P815 cells were used as target and bystander cells, respectively. Antibody cocktail targeting integrin β1, β2 and β7 (anti-ITGB) were applied in (**a**). Twenty randomly chosen NK trajectories are shown in (**a**). Analyses of persistence and velocity from three donors are shown in (**b** and **c**). **(d–f)** An antibody against LFA-1 α chain (anti-ITGAL) was applied. Twenty randomly chosen NK trajectories are shown in (**d**). Analyses of persistence and velocity from three donors are shown in (**e** and **f**).

As NK cells clearly make contact with bystanders (shown in Fig. 2e), we examined whether cell adhesion molecules on NK cells are involved in the decreased search time. Integrins are essential for NK cell migration^24^,^25^,^26^. From the expression profile of integrins in NK cells determined by microarrays (Supplementary Table 1), we found that integrin β1, β2 and β7 are the three dominantly expressed integrin β-chains in NK cells. We thus applied a cocktail of antibodies targeting β1, β2 and β7 integrin to functionally block the interaction of integrins with other effector molecules^27^,^28^,^29^. The migration persistence was impaired by the elimination of β-integrin function as shown in the trajectories and the analysis (Fig. 5a,b), whereas the accelerated NK cell migration by bystander cells was not affected (Fig. 5c).

The other predominantly expressed integrin identified by microarray analysis is integrin α-L chain (ITGAL, Supplementary Table 1), the distinct α chain for lymphocyte function-associated antigen 1 (LFA-1) involved in asymmetrical NK cell spreading and migration^30^. A neutralizing antibody targeting LFA-1 α chain (ITGAL) changed neither the velocity nor the persistence of NK cells in the presence of bystander cells (Fig. 5d–f). Taken together, bystander-enhanced NK velocity is regulated by H_2_O_2_; whereas bystander-enhanced NK persistence is regulated by β-integrins (β1, β2 and β7) not by the LFA-1 α chain on NK cells.

## Discussion

NK cell migration velocity is enhanced in the vicinity of non-target bystander cells, which increases NK cell dependent target cell killing. This effect is mediated by H_2_O_2_ production of bystander cells. In addition bystander cells enhance NK cell migration persistence in an integrin dependent manner in a 2D but not 3D environment.

An obvious important question is whether bystander cells can promote killing efficiency *in vivo*. Unfortunately, this cannot easily be resolved; however, our results indicate that potentially, all H_2_O_2_-producing non-target cells could act as bystanders. Leukocytes produce large amounts of ROS to fight pathogens at inflammation sites^31^, making them good candidates as bystander cells. We indeed found that H_2_O_2_-producing monocytes can enhance killer cell velocity. In addition, HUVEC, a human endothelial cell line, is able to act as bystander cells (Supplementary Fig. 2) and that P815 cells, a mastocytoma cell line, enhance both NK migration and killing efficiency. Together, these data suggest that the bystander effect is a general phenomenon. Considering that H_2_O_2_ enhances leukocyte recruitment to wounded sites *in vivo* in zebrafish^15^ and that bystanders increase migration velocity in human NK cells in a 3D matrigel environment, it appears likely that the bystander effect operates *in vivo* in humans.

Our hypothesis is that a local increase in killer cell motility, when in contact with or close to bystander cells, is sufficient to decrease search time. This is experimentally very difficult to verify. Since theoretical models have been proven to be a powerful tool to untangle complex cell behavior from experimental data^32^, we analyzed three mathematical models for random search in a two-dimensional confined area. Although upon first examination it appears counterintuitive that without providing a directional cue towards target cells, local acceleration of NK cells in the vicinity of bystander cells can result in a more efficient search process, the models predict that search time decreases systematically with increasing density of bystander cells and with increasing local acceleration. The explanation for this is that in a random search the ensemble of possible search trajectories is not changed by local accelerations and thus the net search time only depends on the velocity along those trajectories. However, bystander cells themselves also represent disk-like obstacles of radius R and we have demonstrated that diffusion in an archipelago of obstacles is slowed down, depending on the density of obstacles, see also^33^. Consequently, there is a competition between diffusion acceleration in the vicinity of bystander cells and the diffusion slowing down due to the bystanders themselves being obstacles. As we have shown the first effect wins when the acceleration region around bystanders and the increased diffusion constant is sufficiently large in these regions and a local motility enhancement through the bystanders is indeed sufficient to increase killing efficiency.

H_2_O_2_ can be generated as a by-product of cellular metabolism^34^,^35^,^36^ or more specifically by specialized enzymes like NADPH oxidases^37^,^38^. Increased ROS production has been observed in various tissues, for instance lung tissues^39^, hepatocytes^40^ and kidney^41^. Studies also indicate that in the kidney NADPH oxidases are expressed in a regional and cell-specific manner^41^. Thus it is expected that the bystander effect depends on the microenvironment generated by the interaction of tumor or inflammation with a specific organ or tissue. This in turn suggests that in different tissues or microenvironments of cancer or inflammation we could have different cell types as bystanders. On the other hand, too much H_2_O_2_ in the microenvironment, i.e. higher than 10 μM, induces the apoptosis of NK cells, especially in cytotoxic CD56^dim^ NK cells^42^,^43^. Therefore appropriate H_2_O_2_ levels may be of great importance to optimize the effect of immune surveillance.

Our results show that in 2D bystander cells enhance both velocity and persistence of NK cells (Fig. 2b,c). However, in a 3D scenario, only the velocity of NK is increased by the presence of bystander cells (Fig. 2i). It is reported that *in vivo*, the motility of murine leukocytes is largely independent of integrin^44^,^45^. Together, this suggests that in 3D, primary human NK cells could also employ more integrin-independent amoeboid movement.

We have identified that β1, β2 and β7 integrins on NK cells are responsible for regulating the bystander-enhanced NK persistence. Since we showed that H_2_O_2_ is involved in the bystander-enhanced velocity of NK migration but not in the persistence change, it is unlikely that integrins on NK cells are the effector molecules of environmental H_2_O_2_. There is evidence that in CD4^+^ T cells ROS can regulate several molecules in T cell receptor signaling pathways including protein tyrosine phosphatase, Src-family kinases^23^ and Orai1 channels^46^. These proteins may be promising candidates to mediate the H_2_O_2_-dependent increase in NK cell migration velocity. Regardless of the molecular mechanism of H_2_O_2_-mediated bystander-enhanced migration and killing efficiency of NK cells, H_2_O_2_ is an important factor how the microenvironment influences tumor progression and elimination by killer cells.

## Materials and Methods

### Reagents and antibodies

All chemicals not specifically mentioned were from Sigma (highest grade). Calcein-AM, CellMask™ Orange, CellTracker™ Deep Red, CellTrace^TM^ and calcein red-orange AM were purchased from ThermoFisher Scientific. The antibodies used in our experiments include: antibody against Integrin-α-L (ITGAL, LFA-1) (Antibodies-online GmbH), human Integrin β1 (R&D Systems) and human Integrin β2 (R&D Systems) polyclonal antibodies and antibody against human/mouse Integrin β7 (Biolegend). Polybead^®^ Polystyrene Microspheres (15 μm, 18328–5) were purchased from Polysciences. Cultrex^®^ Reduced Growth Factor Basement Membrane Extract and PathClear^®^ was purchase from Trevigen.

### Cell culture

Raji, K562 and P815 cells were cultured in RPMI-1640 medium (ThermoFisher Scientific) supplemented with 10% FCS at 37 °C with 5% CO_2_. Peripheral blood mononuclear cells (PBMC) were obtained from healthy donors as previously described^19^. Primary human NK cells and monocytes were negatively isolated from PBMC from the same donors using Dynabeads^®^ Untouched™ Human NK Cells Kits and Dynabeads^®^ Untouched™ Human Monocytes Kits, respectively (ThermoFisher Scientific). Before use, the isolated primary NK cells and monocytes were cultured overnight at 37 °C with 5% CO_2_ in AIMV medium with 10% FCS at a density of 2 × 10^6^ and 1.5 × 10^6^ cells/ml, respectively.

### Real time killing assay

The real-time killing assay was carried out as described before^19^. Briefly, target cells were loaded with calcein-AM and plated on a Falcon^®^ 96-well black with clear flat bottom plates (Corning) at a density of 2.5 × 10^4^ cells/well. NK cells were subsequently added and the fluorescence was measured by a GENios Pro micro-plate reader (TECAN) every 10 minutes for 4 hours at 37 °C. Bystander cells were plated together with target cells.

### Time-lapse imaging and migration analysis

Target cells were loaded with calcein-AM and bystander cells were loaded with CellTrace calcein red-orange-AM. Afterwards 75 × 10^3^ bystander cells and 25 × 10^3^ target cells were plated for each well in 96-well plates. 1.25 × 10^5^ NK cells were then added to each well. NK cell migration was visualized at 37 °C using the cell observer microscope (Zeiss) equipped with a 10× objective (Fluar, NA 0.50). The green channel (Ex 470 nm/Em 525 nm) and the red channel (Ex 550 nm/Em 600 nm) as well as wide field images were acquired at an interval of 16.8–38.2 seconds according to the experimental conditions. Cells were tracked manually with ImageJ 1.45 s plug-in Speckle TrackerJ. Immobile NK cells were discarded from statistical analysis. For testing the function of adhesion molecules, NK cells were pre-incubated with the neutralizing antibody or vehicle at 37 °C for 30 minutes before being added to target cells. The cocktail of antibodies against integrins contains 10 μg/ml anti-integrin β1, 20 μg/ml anti-integrin β2 and 10 μg/ml anti-integrin β7. The antibody against LFA1 was used at a concentration of 10 μg/ml.

### 3D live cell imaging with lightsheet microscopy

Target K562 cells were loaded with calcein-AM, bystander cells (monocytes) were loaded with CellMask™ Orange, and NK cells were loaded with CellTracker™ Deep Red. Target cells and NK cells were mixed in AIMV with 10% FCS with or without bystander cells. Afterwards, cell suspension was mixed with growth factor reduced Matrigel to an end concentration of 8.1 mg/ml. This cell/matrigel mix was transferred into a capillary (BRAND) and kept at 37 °C with 5% CO_2_ for 30 minutes for polymerization. Final density of NK cells, bystander monocytes and target K562 cells were 1 × 10^7^, 1 × 10^7^ and 5 × 10^6^ cells/ml, respectively. Afterwards, the migration of cells was visualized with the lightsheet micoscopy (5× objective) at 37 °C with a z-step size of 1 μm and time interval of around 30 seconds. NK cells were tracked and analyzed using Imaris 8.1.2 (containing Imaris, ImarisTrack, ImarisMeasurementPro, ImarisVantage; Bitplane AG, software available at http://bitplane.com).

### Amplex UltraRed Assay

To determine the concentration of H_2_O_2_ generated during the killing process, we carried out a direct peroxidase assay using Amplex UltraRed (ThermoFisher Scientific). Target cells (2.5 × 10^4^) alone or together with bystander cells (7.5 × 10^4^) were seeded in 96-well plates in 100 μl phenol red free DMEM-F12 supplemented with 10 mM HEPES. Twenty minutes after the cells were seeded, NK cells in 10 μl DMEM-F12 medium were added carefully on top of the pre-seeded cells. DMEM-F12 alone was used as the background control. Freshly mixed 100 μM Amplex UltraRed regent containing 10 units/ml SOD and 0.1 units/ml HRP ± 10 units/ml catalase in the phenol red free DMEM were added into wells immediately after the NK cell addition. Plates were immediately measured with a Genios Pro micro-plate reader (TECAN) at an excitation/emission of 535 nm/590 nm at 37 °C every 4 or 6 minutes for 4 hours.

### Degranulation assay

NK cells were settled in 96-well plates with target cells and/or bystander cells as indicated in the figures. Cells were kept in AIMV medium containing Alexa^488^ conjugated anti-human CD107a antibody (Biolegend) and GolgiStop^TM^ (BD Biosciences). Cells were incubated at 37 °C with 5% CO_2_ for 4 hours. Afterwards cells were washed twice and stained with APC/Cy7 conjugated anti-human CD16 Antibody (Biolegend) to identify NK cells. Data were acquired using a FACSVerse™ flow cytometer (BD Biosciences) and were analyzed with FlowJo v10 (FLOWJO, LLC). Only CD16^+^ cells in lymphocytes population were gated for further analysis.

### Microarray measurement and data analysis

Preparation of the templates and microarray analysis were performed as described before^47^. Using the Agilent Feature Extraction Software the raw values from the scanned image files were extracted. In the next step, the R software was used to normalize the data (quantile normalization) and to perform a log2 transformation. The normalized data were used to compare the expression differences between the different samples.

### Computational analysis of the mathematical model

A kinetic Monte Carlo scheme was implemented to generate the Brownian motion of disk-like particles in a square geometry; disks represent targets, obstacles, and bystanders. A very efficient First Passage Kinetic Monte Carlo algorithm (FPKMC) was used as documented in the literature^48^,^49^,^50^, also denoted as GFRD MC (Greens function reaction diffusion Monte Carlo). For each parameter set 40.000 samples of the random trajectories and the random times were generated when targets were hit and removed. Movies 2 and 3 in the Supplementary Material show example trajectories for the generated search processes without and with bystanders, respectively. A subsequent average over these 40.000 samples produced the results for the average number of remaining targets at time t, <N_t_(t) >, for each parameter set, which is presented and discussed in the main text.

### Statistical analysis

Data are presented as mean ± S.E.M unless otherwise indicated. The differences between two groups were tested using the student’s t-test. For multiple comparisons between groups, the significance was first examined using the One way ANOVA test, followed by Kruskal-Wallis test. *P < 0.05; **P < 0.01; ***P < 0.001; n.s., not significant.

### Ethical considerations

Research carried out for this study with human material (leukocyte reduction system chambers from human blood donors) is authorized by the local ethic committee (declaration from 16.4.2015 (84/15; Prof. Dr. Rettig-Stürmer)).

## Additional Information

**How to cite this article**: Zhou, X. *et al*. Bystander cells enhance NK cytotoxic efficiency by reducing search time. *Sci. Rep.* **7**, 44357; doi: 10.1038/srep44357 (2017).

**Publisher's note:** Springer Nature remains neutral with regard to jurisdictional claims in published maps and institutional affiliations.

## Supplementary Material

Supplementary Movie 1

Supplementary Movie 2

Supplementary Movie 3

Supplementary Information
