# Supplementary material for: Bystander cells enhance NK cytotoxic efficiency by reducing search time
Source: Sci Rep. 2017 Mar 13;7:44357. doi: 10.1038/srep44357 (PMC5347013; doi:10.1038/srep44357)
Supplement: Supplementary Information [file srep44357-s4.pdf]

Bystander cells enhance NK cytotoxic efficiency by reducing search time

Xiao Zhou[1], Renping Zhao[1], Karsten Schwarz[2], Matthieu Mangeat[2,4], Eva C. Schwarz[1], Mohamed Hamed[3,5], Ivan Bogeski[1], Volkhard Helms[3], Heiko Rieger[2], Bin Qu[1]

Supplementary Information

Lattice based models

We considered two lattice based reaction-diffusion models with discrete time-steps Δt in two dimensions including four different types of particles: searchers (active NK cells, N_k), targets ($N_t(0)$), obstacles (inactive NK cells, N_o) and bystanders (N_b). The simulations took place on a 60×60 square lattice (and its dual lattice, see discrete Model 1 and 2, Supplementary Fig. 3a, b) with periodic boundary conditions and simple exclusion (no/one particle per lattice side). The lattice size was checked to be large enough to avoid finite size effects at particle densities $(N_k+N_t(0)+N_o+N_b)/(60\times60)$. In both investigated models, the searchers, targets and obstacles share the same lattice.

In the discrete Model 1 the bystanders are also placed on this lattice and all cells could move. Bystanders and killers change their positions, if a step in the direction of a neighboring NK cell or bystander is proposed. In the discrete Model 2, the bystanders are always immobile and placed between the lattice as shown in Supplementary Fig. 3b. In both models a target is found by an NK cell when they are located at the same lattice side at the same time. In this case, the target is considered killed and thus disappears, whereas the NK cell continues with its movement. The normal hopping probability of a mobile particle is

given by p/4 (usually p=1/2) in each of the four directions. A special role is always assigned to the bystanders, as they increase the hopping probability in their nearest neighborhood.

Several different scenarios were investigated for the increased hopping rates of the stochastic processes in both models:

Model 1: Different combinations for the motility of the particle types were investigated as listed in Supplementary Fig. 3c. Particles (searchers and obstacles) which are accelerated by bystanders increase their hopping rate to an allowed direction to q/4 (with q>p). In Supplementary Fig. 3c the lower panel shows the half time $t_{1/2}$ for different setups as a function of the number of bystanders N_b.

Model 2: Targets and bystanders are immobile in this model. Searchers and obstacles are accelerated by bystanders. Several different scenarios for the acceleration as depicted in Supplementary Fig. 3d were investigated. Their half time $t_{1/2}$ is shown as a function of number of bystanders (Supplementary Fig. 3d).

In both models, three update schedules for the particles have been applied: a sequential ordered update, a sequential update with stochastic order and a totally randomized choice of particles. The percentage of the targets found by NK cells, $1 - \langle N_t(t)\rangle / N_t(0)$, from the discrete Model 1, shows that the more bystander cells, the more target cells are found by NK cells (Supplementary Fig. 3e). For the same model and the same parameters, the half time $t_{1/2}$ as a function of q decreases with an increase of bystander cell numbers (Supplementary Fig. 3f). These results are in good agreement with the results from a continuous model shown in Fig. 3e and g, respectively.

In conclusion, the discrete model also predicts that locally accelerated migration of killer cells by bystander cells is able to decrease the target search time and thus increase the efficiency of target elimination.

Supplementary figures and tables

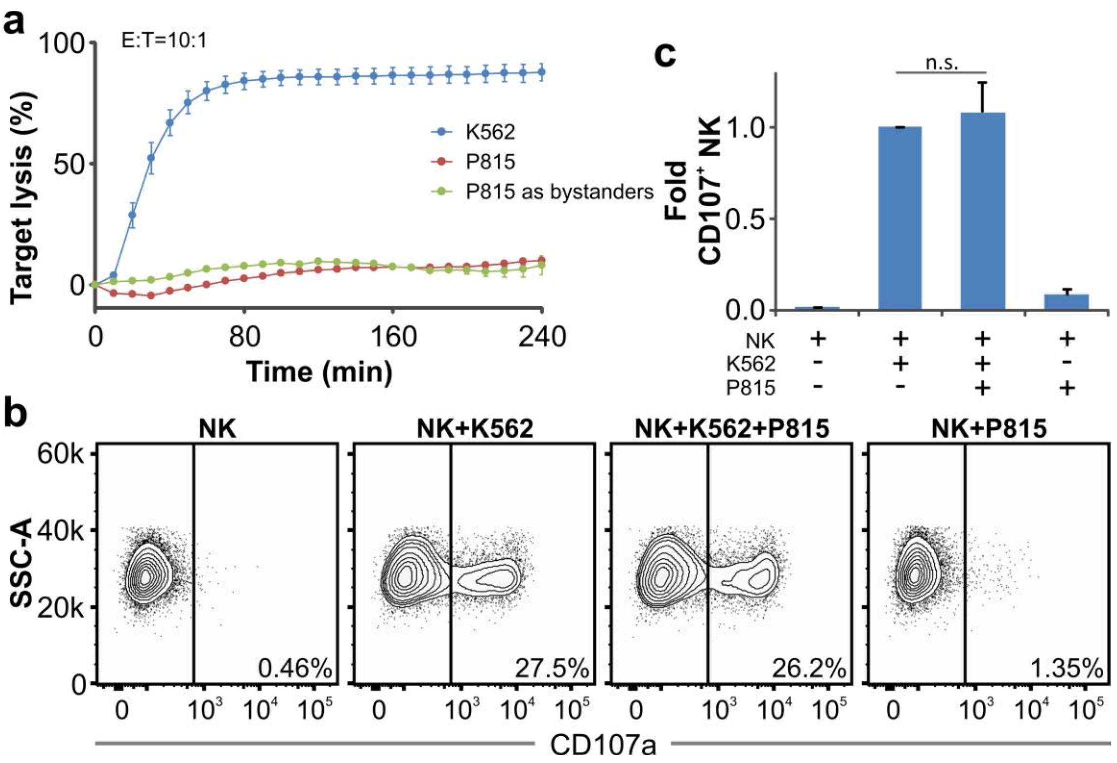

Supplementary Fig. 1 The presence of bystander P815 cells does not alter lytic granule release in NK cells upon target recognition. (a) P815 cells cannot be killed by primary human NK cells. K562 and P815 cells were loaded with calcein-AM, respectively. K562 cell alone (blue, 25×10^3 cells/well,), P815 cell alone (red, 25×10^3 cells/well) or P815 as bystander (green, 75×10^3 cells/well of calcein-loaded P815, 25×10^3 cells/well of unloaded target K562 cells) were incubated with 25×10^4 cells/well primary human NK cells at an E:T ratio of 10:1. The lysis of calcein-loaded cells was determined by the real-time killing assay. **(b, c)** Degranulation of NK cells is not changed by the presence of bystander P815 cells. K562 cells and P815 cells were used as targets and bystander cells respectively, with primary NK cells. The fold change in LG-released NK cells is shown in **c** (n = 3). The level of LG-released NK cells in the condition of NK+K562 was set to 1.

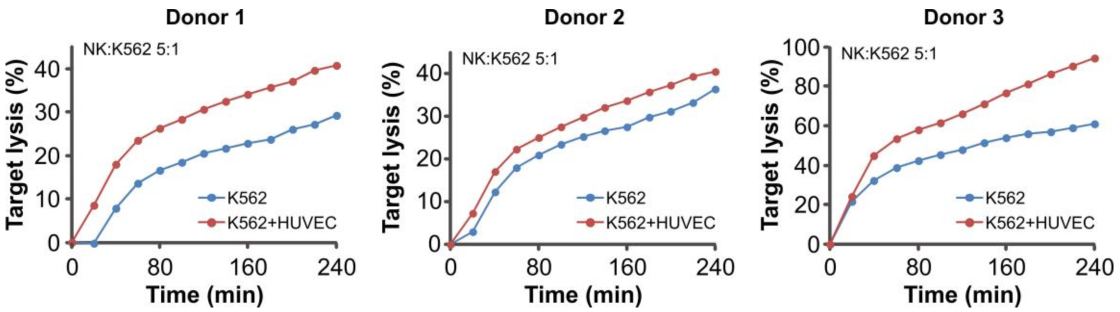

Supplementary Fig. 2 HUVEC cells as bystanders increase NK killing efficiency. K562 were loaded with calcein-AM and plated at a density of 25×10^3 cells/well. K562 cell alone (blue) or together with 75×10^3 cells/well bystander HUVEC cells (red) were incubated with 12.5×10^4 cells/well primary NK cells isolated from three healthy donors.

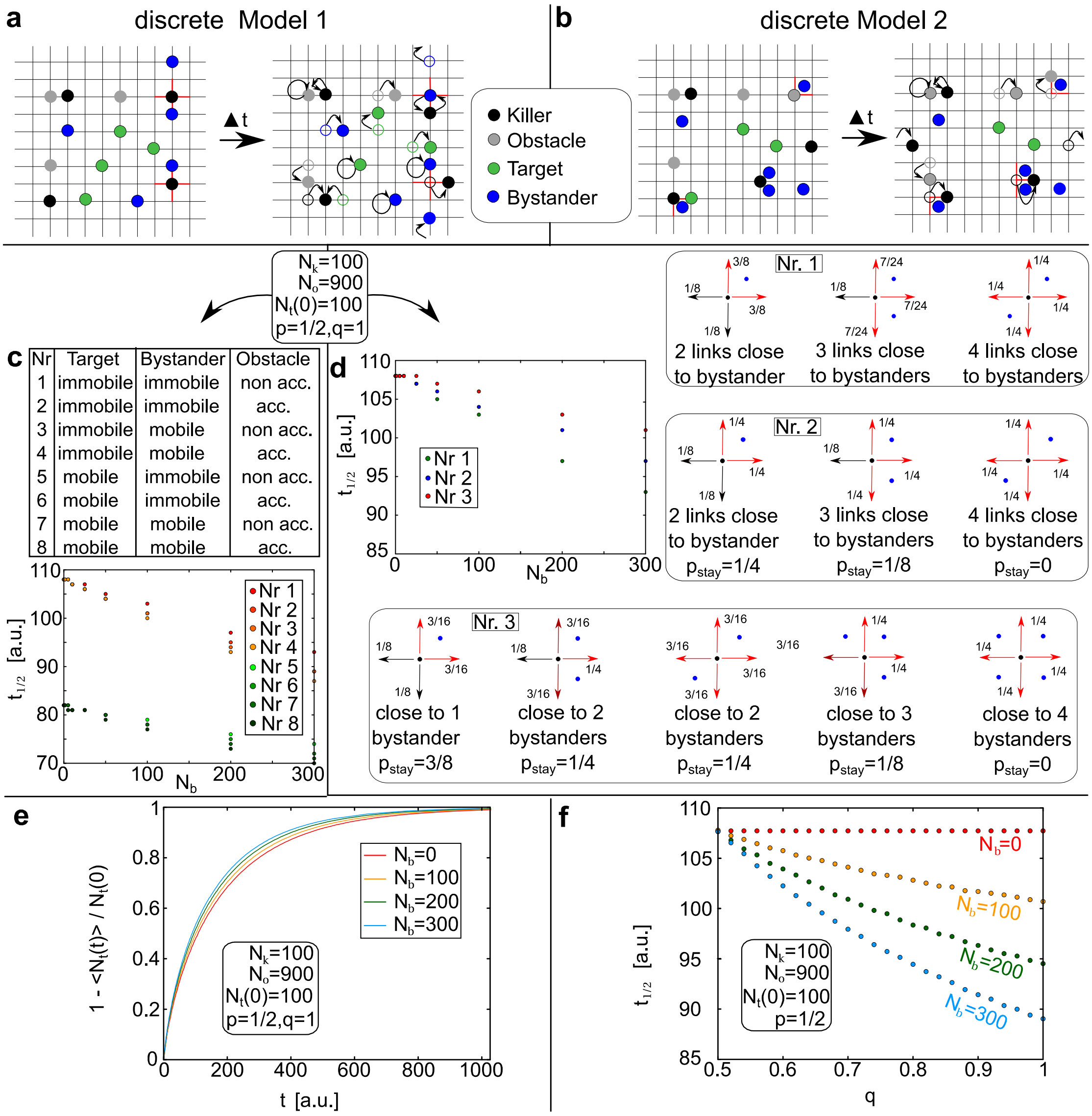

Supplementary Fig. 3 Lattice based mathematical model simulating the migration of NK cells in the presence of bystander cells. (**a**) Sketch of the stochastic process for the discrete Model 1: searchers, targets, obstacles and bystanders are placed on the same periodic lattice. With probability p/4, a particle will hop to its neighbor spot, if there is no other particle. In the neighborhood of obstacles the hopping probability is increased to q/4 (indicated in red). (**b**) Sketch of the stochastic process for the discrete Model 2: searchers, targets and obstacles are placed on the same periodic lattice, immobile bystanders on the corresponding dual lattice. With probability p/4, a particle will hop to its neighbor's spot, if there is no other particle. In the neighborhood of obstacles the hopping probability is increased (indicated in red). (**c**) Half time $t_{1/2}$ as a function of the number of bystanders for different update scenarios in Model 1. (**d**) Half time $t_{1/2}$ as a function of the number of bystanders for different update scenarios in Model 2. (**e**) Percentage of targets found by NK cells as a function of time for N_k=100 killers, N_o=900 obstacles, $N_t(0)$=900 targets and different numbers of bystanders N_b on a 60×60 square lattice (corresponding to a maximum occupancy of 3600 cells). **(f)** The half time $t_{1/2}$ as a function of the acceleration probability q>p=1/2 for the same particle numbers as in **e.**

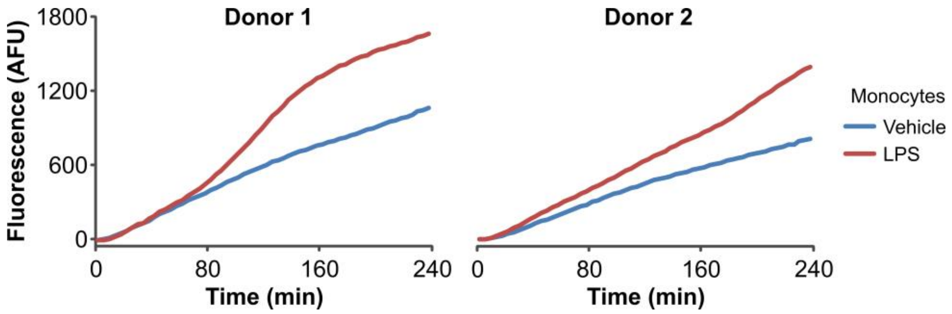

Supplementary Fig. 4 The production of H_2O_2 by primary human monocytes. Using the Amplex UltraRed assay we assessed H_2O_2 production by primary peripheral monocytes from two healthy donors. Primary human monocytes were treated with vehicle or 10 ng/ml Lipopolysaccharide (LPS) for 24 hours and plated at a density of 7.5×10^4 cells/well. The production of H_2O_2 was detected at Ex/Em of 535/590 nm every 6 minutes for 4 hours by the microplate reader.

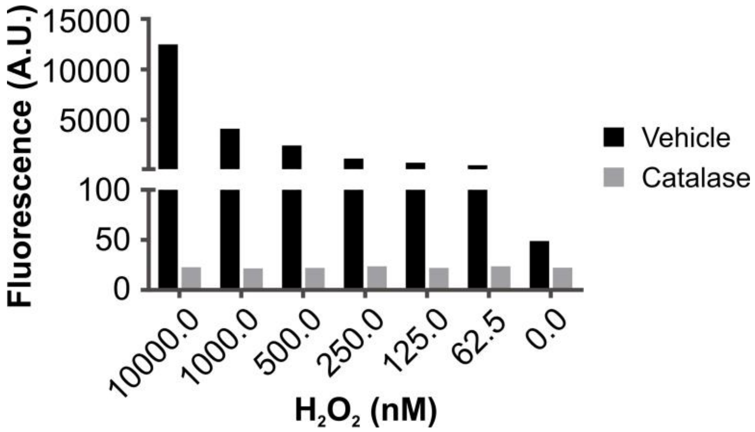

Supplementary Fig. 5 Environmental H_2O_2 is depleted by catalase. Catalase (10 units / ml) or vehicle (PBS) was added to the corresponding wells. Then various concentrations of H_2O_2 were introduced into the phenol-red free DMEM/F12 medium as indicated. Amplex UltraRed was given directly after addition of H_2O_2. Mean of the triplicates for each condition is shown.

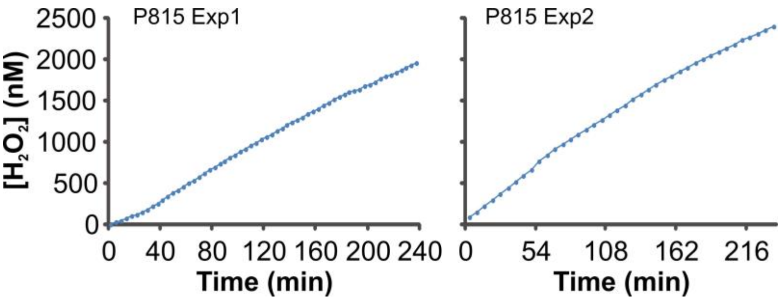

Supplementary Fig. 6 P815 bystander cells produce H_2O_2. As for real-time killing assay, P815 cells were plated as 75×10^3/well. Concentrations of H_2O_2 were determined by the Amplex UltraRed assay. Fluorescence (ex/em: 535/590 nm) was measured at 37°C every 4 or 6 minutes for experiment 1 and 2, respectively.

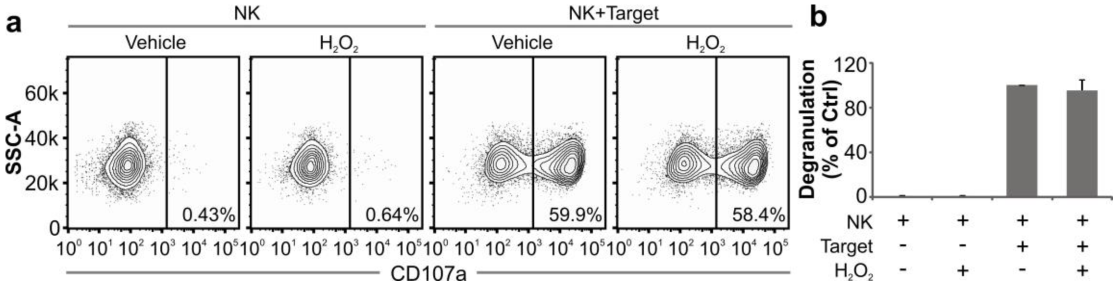

Supplementary Fig. 7 The degranulation of killer cells is not affected by H_2O_2 (2μM). K562 cells were used as target cells with an E:T ratio of 10:1. One representative donor out of three is shown in **a**. Quantification of all three donors is shown in **b**.

Genes	mean.NK	sd.NK
NM_133376 ITGB1	9.7	0.1
NM_000211 ITGB2	14.6	0.2
NM_000212 ITGB3	2.3	1.3
NM_000213 ITGB4	5.1	0.4
NM_002213 ITGB5	2.0	0.7
NM_000888 ITGB6	0.9	0.1
NM_000889 ITGB7	14.2	0.3
NM_002214 ITGB8	0.7	0.0
NM_002209 ITGAL	14.3	0.2
NM_181501 ITGA1	1.8	1.2
NM_000419 ITGA2B	4.6	0.3
NM_002203 ITGA2	1.1	0.1
NM_002204 ITGA3	4.0	0.5
NM_000885 ITGA4	10.0	0.2
NM_002205 ITGA5	10.2	0.2
NM_000210 ITGA6	7.9	0.8
NM_002206 ITGA7	8.8	0.3
NM_002207 ITGA9	5.8	0.4
NM_003637 ITGA10	8.9	0.2
NM_001004439 ITGA11	4.4	0.3

Supplementary Table 1 Analysis of mRNA expression of integrin family proteins using microarray data. The mRNA expression of integrin family proteins was analyzed using microarray data from NK cells from eleven donors. Results are presented as mean ± S.D. The fluorescence values were quantile normalized and logged to the base 2 (log2). The dynamic range of the data is from $2^{0.704}$ (=1.629) to $2^{18.4}$ (=345901).

Movie legends

Supplementary Movie 1. NK cell migrates from one bystander to the next until it finds a target cell. Migration of primary human NK cells was visualized at 37°C every 20 seconds for 1 hour with the cell observer. Target K562 cells were loaded with calcein-AM. Bystander P815 cells were loaded with calcein red-orange-AM. Non-labeled cells are NK cells. Scale bar is 30 μm.

Supplementary Movie 2. Monte Carlo sample without bystanders. Similar to Fig. 3a, NK cells are shown in black, obstacles are grey, and targets are green. If a target is found by a killer it fades away and the killer continues its motion. The red circles, sectors and rectangles (here called protection boxes) explain the numerical simulation method. Within these protection boxes, it is possible to sample fully correct (no approximation via discretization) the first passage probability in time to the boundary and a corresponding first passage position [1-3]. By choosing the boxes in a way that the distance to targets and obstacles is always larger than R, it is possible to propagate particles in large steps within the whole box, without losing precision. Hence, the algorithm always adapts its step-size to the local density of its surrounding. By doing so, it is possible to fight the bottleneck of simulating diffusion within different length scales. The chosen parameters are $N_k=N_t(0)=20$, $N_o=200$, $N_b=0$ and $r_{obs}=R=0.5$, i.e. the sample belongs to the dark blue line in Fig. 3b and to the green dot at $N_o=200$ in Fig 3c.

Supplementary Movie 3. Monte Carlo sample with bystanders. In addition to the caption of Movie 2, there are $N_b=50$ bystanders, which are shown in dark blue. The light blue ring surrounding the bystanders is the area of accelerated diffusion, with $D_{acc}=4$ and $\Delta=3$. The

other parameters are identical to Movie 2 (N_k=N_t(0)=20, N_o=200 and r_{obs}=R=0.5), i.e. the sample belongs to the dark blue line in Fig. 3e, to the red dot at N_b=50 in Fig 3f, and to the dark blue dot at D_{acc}=4 in Fig. 3g.

Supplementary references

1 Oppelstrup, T. *et al.* First-passage kinetic Monte Carlo method. *Phys Rev E Stat Nonlin Soft Matter Phys* **80**, 066701, doi:10.1103/PhysRevE.80.066701 (2009).

2 van Zon, J. S. & ten Wolde, P. R. Green's-function reaction dynamics: a particle-based approach for simulating biochemical networks in time and space. *J Chem Phys* **123**, 234910, doi:10.1063/1.2137716 (2005).

3 Schwarz, K. & Rieger, H. Efficient kinetic Monte Carlo method for reaction-diffusion problems with spatially varying annihilation rates. *J Comput Phys* **237**, 396-410, doi:10.1016/j.jcp.2012.11.036 (2013).
